# The Efficacy of Lactocare® Synbiotic on the Clinical Symptoms in Patients with Psoriasis: A Randomized, Double-Blind, Placebo-Controlled Clinical Trial

**DOI:** 10.1155/2022/4549134

**Published:** 2022-10-07

**Authors:** Ali Akbarzadeh, Pedram Alirezaei, Amin Doosti-Irani, Maryam Mehrpooya, Fatemeh Nouri

**Affiliations:** ^1^Department of Pharmaceutical Biotechnology, School of Pharmacy, Hamadan University of Medical Sciences, Hamadan, Iran; ^2^Psoriasis Research Center, Hamadan University of Medical Sciences, Hamadan, Iran; ^3^Department of Epidemiology, School of Public Health and Research Center for Health Sciences, Hamadan University of Medical Sciences, Hamadan, Iran; ^4^Department of Clinical Pharmacy, School of Pharmacy, Hamadan University of Medical Sciences, Hamadan, Iran

## Abstract

**Background:**

Attention to the administration of probiotics for the treatment of psoriasis has recently increased.

**Aim:**

In the present study, improvements in dermatology life quality index (DLQI), psoriasis area severity index (PASI), and visual analogue scale (VAS) scores in the psoriasis patients receiving Lactocare® probiotic were compared to psoriasis patients receiving placebo.

**Methods:**

A total of 52 psoriasis patients were included in this study and randomly divided into treatment and placebo (control) groups. The control group received topical hydrocortisone associated with placebo; in the treatment group, Lactocare® was administrated orally associated with hydrocortisone. The mean of VAS, DLQI, and PSAI scores was recorded and evaluated pretreatment and post-treatment in both groups for 3 months. The mean of the scores in the control groups was compared to the treatment group. Intragroup analysis was preformed with a comparison of the mean of these scores at baseline 4-, 8-, and 12-weeks post-treatment.

**Results:**

In the treatment group, a significant decrease was seen in PASI, VAS, and DLQI scores compared to the control group on week 12 post-treatment.

**Conclusion:**

Oral administration of Lactocare® probiotic (two times daily) associated with administration of topical hydrocortisone resulted in the improvement of PASI, DLQI, and VAS scores in the patients with psoriasis after 12 weeks of treatment. PASI reduction occurred in all patients who received probiotics.

## 1. Introduction

Psoriasis is a chronic, inflammatory, and immune-mediated disease characterized by the development of scaly, sharply demarcated erythematous, pruritic, indurated, and often painful skin plaques [1]. It is estimated that more than 125 million (0.09–11.4%) people suffer from psoriasis in the world. Psoriasis not only is considered to be a dermatological disease but has also recently been defined as a systemic one due to the multiple organs' involvement with an important impact on social, psychological, and economic life [2]. Although the pathogenesis of psoriasis has not yet been completely known, the interaction between innate and adaptive immune systems is known as the underlying pathomechanisms of psoriasis [3]. In addition, genetic background, ethnicity, and environmental factors have been shown to affect the onset of the disease [3, 4]. The inflammatory cytokines, such as interleukin IL-12, IL-17, IL-23, and tumor necrosis factor TNF-*α* are elevated in the peripheral blood of patients with psoriasis [5].

Topical and systemic immunosuppressants (systemic corticosteroids), cyclosporine or methotrexate, and phototherapy are used for the treatment of psoriasis [6]. Anti-TNF-*α*, anti-IL-23, and IL-17, and monoclonal antibodies have been recently developed for the treatment of the disease [7, 8]. Despite the fact that monoclonal antibodies are more effective than topical or systemic immunosuppressants, they are associated with high economic costs and adverse effects [9]. Clinicians have recently been attempting to develop better treatment options for skin illnesses such as psoriasis, and probiotic therapy is gaining popularity [10]. In this regard, some studies have been conducted on integrative remedies for skin diseases such as topical cream of the *Malva sylvestris* extract [11], oral supplementation of whey protein [12], and other natural compound [13] formulations that significantly improve the severity of inflammatory skin diseases. Probiotics are live exogenous nonpathogenic microorganisms that confer a health benefit for atopic dermatitis, psoriasis, and acne when they are administered in sufficient quantities [14, 15]. Probiotics have been reported to protect the host from infections resulting from the immunomodulatory effect, increasing the production of immunoglobulins, and activation of immune cells such as mononuclear cells and lymphocytes [16]. Probiotics have been hypothesized to have a beneficial role in the treatment of psoriasis. Since the anti-inflammatory effect of a brand of preprobiotics named Lactocare® on the disease severity of ulcerative colitis patients [17] and also on the serum electrolytes and trace elements in psoriatic patients [18] has previously been reported, this study aimed to evaluate the effect of Lactocare® on the treatment of psoriasis using the psoriasis area severity index (PASI), Visual Analogue Scale (VAS), and dermatology life quality index (DLQI).

## 2. Material and Methods

### 2.1. Subject and Study Design

In this case-control double-blind clinical trial study (this study is registered by the Iranian Registry of Clinical Trials with the code: IRCT20120215009014N323) that was conducted during 2020–2021, and the required sample size with a standard deviation of 1.5 units, the first type of error of 5%, and the test power of 80% were considered in two groups of 32 people.

### 2.2. Intervention and Ethics

After providing the written informed agreement, 52 individuals with psoriasis were enrolled in this study (27 and 25 patients in the placebo and therapy groups, respectively) (12 people lost owing to the COVID-19 pandemic, and the study was discontinued). The Ethics Committee of Hamadan University of Medical Sciences, Hamadan, Iran, also approved the study protocol (ethical approval number: IR. UMSHA. REC. 1398.725).

### 2.3. Outcomes and Measures

The inclusion criteria were a clinical diagnosis of psoriasis confirmed by a dermatologist, aged between 18 and 60 years and a lack of history of consumption of probiotics and drugs 1 to 6 weeks before the beginning of the experiment, PASI ≥2%. Diabetic and immunosuppressant patients and patients with a history of immunosuppressing drug consumption were excluded from the study. The severity of the disease was measured using the PASI score. In brief, the body was classified into four regions including the head, trunk, and upper and lower extremities. To calculate the PASI score, the doctor graded the psoriasis plaques found on each body region for their combined redness, thickness, and scaliness (A scores). The severity of the plaques in each region is graded on a 0 to 4 scale, with 0 meaning no involvement and 4 meaning severe involvement. Next, he calculated the amount of the surface area on each body region that is covered by plaques (B scores). The total surface area affected by psoriasis is graded from 0 to 6, with 0 meaning no involvement and 6 meaning greater than 90 percent of the region covered in plaques. Then, he multiplied the A score by B score for each body region to get four C scores. Then, he multiplied each C score by the amount of the body surface area that the region represents. This amount is 0.1 for the head and neck, 0.2 for the arms, 0.3 for the trunk, and 0.4 for the legs.

DLQI is a self-reported questionnaire that consists of 10 questions to evaluate the effect of psoriasis on the quality of life of patients with psoriasis [19]. This 4-point scale (0 = not at all to 3 = very much) questionnaire was divided into six commonly identified categories. The highest possible DLQI score was 30, and the higher scores indicate a higher effect on the quality of life [20]. The patients with psoriasis were asked to report their itch VAS score during the last 24 h on a VAS score ranging from “no itch at all” to “worst (maximal intensity) itch you can imagine. The patients were randomly divided into two groups including the control and treatment groups via block randomization. The control group received weak corticosteroid (hydrocortisone) topically associated with placebo for three months. In the treatment group, Lactocare® purchased from Zist Takhmir Company, (two times daily) was administrated orally associated with weak corticosteroid or hydrocortisone for three months. VAS, DLQI, and PSAI scores were evaluated pre-treatment and post-treatment. The mean of the scores in the control groups was compared to the treatment group.

### 2.4. Statistical Analysis

Data were analyzed using SPSS version 22, and the data were expressed as the mean ± standard error of the mean (SEM). The mean of the scores in both groups was compared using an independent *t-*test. Intragroup analysis was conducted using repeated-measure *ANOVA*. The chi-squared test was used to compare the demographic data, and one-way ANOVA was used to compare the mean scores in various age groups. *P* < 0.05 was considered a statistical difference.

## 3. Results

### 3.1. Demographical Findings

A total of 52 patients (33 male and 19 female) completed the study. The male-to-female ratio was 1.73 in the psoriasis patients. Demographical data including sex, duration of disease, and age are shown in Table 1. There was no significant difference between the control and treatment groups in sex (*P* > 0.05). There was no significant difference between the treatment and control groups in terms of the mean duration time of (*P* > 0.05) (Table 1).

### 3.2. PASI, VAS, and DLQI Findings on Placebo Compared to Treatment

The comparison of the baseline, weeks 4, 8, and 12, PASI, VAS, and DLQI scores in both groups was performed to determine the effectiveness of treatment. The results are shown in Table 2. There was no significant difference between the treatment and placebo groups in terms of the baseline PASI score as well as the PASI score of weeks 4 and 8 post-treatment (*P* > 0.05). However, in the treatment group, a significant decrease was seen in the PASI score compared to the control group on week 12 post-treatment (4.08 ± 0.28 vs 6.19 ± 0.39, *P* < 0.001) (Supplementary 1).

There was no significant difference between the treatment and placebo groups in terms of the baseline VAS score as well as the VAS score of weeks 4 and 8 post-treatment (*P* > 0.05). However, in the treatment group, a significant decrease was seen in the VAS score compared to the control group on week 12 post-treatment (32.24 ± 4.29 vs 48.11 ± 4.88, *P*=0.019) (Supplementary 2). There was no significant difference between the treatment and placebo groups in terms of the baseline DLQI score as well as the DLQI score of weeks 4 and 8 post-treatment (*P* > 0.05). However, in the treatment group, a significant decrease was seen in the DLQI score compared to the control group on week 12 post-treatment (6.56 ± 0.63 vs 8.92 ± 0.71, *P*=0.017) (Supplementary 3).

The PASI, VAS, and DLQI scores at the baseline and at weeks 4, 8, and 12 were compared to assess treatment efficacy during the study, and the results are shown in Table 3. In the treatment group, the intragroup analysis showed that the mean PASI, VAS, and DLQI scores were significantly lower at weeks 4, 8, and 12 post-treatment than that at baseline. In addition, the PASI score was significantly lower at week 12 than those of weeks 8 and 4 post-treatment as well as at week 8 than that of 4 weeks post-treatment (*P* < 0.001).

For evaluation of the efficacy of placebo during the study, the PASI, VAS, and DLQI scores in each group at the baseline and at weeks 4, 8, and 12 were compared, and the obtained data are shown in Table 4. In the control group, the intragroup analysis showed that no significant difference was seen between all post-treatment interval times with each other as well as the baseline in terms of PASI, VAS, and DLQI scores (*P* > 0.05).

### 3.3. PASI, VAS, and DLQI Score Findings Based on Age and Sex

Regardless of the treatment, the patients were classified into 4 age groups: 20 to 30 (10 cases, 19.2%), 31 to 40 (17 cases, 32.7%), 41 to 50 (14 cases, 26.9%), and up to 51 (11 cases, 21.2%) years old. The PASI, VAS, and DLQI scores of various ages are shown in Table 5. There was no significant difference between both groups in terms of various age classifications. The frequency of age 20 to 30, 31 to 40, 41 to 50, and up to 51 years old were 20.0% (5 cases), 16.0% (4 cases), 36.0% (9 cases), and 28.0% (7 cases) in the treatment group as well as 18.5% (5 cases), 48.1% (13 cases), 18.5% (5 cases), and 14.8% (4 cases) for the control group, respectively. Regardless, the gender and the mean PASI, VAS, and DLQI scores in various ages of the treatment group are shown in Table 5. There was no significant difference respected in the mean of PASI, VAS, and DLQI scores in various age groups in the treatment group.

There was no significant difference between male and female patients in terms of PASI, VAS, and DLQI baseline and all interval times post-treatment in the treatment group as well as the control group (Supplementary 4.). At the 12-week follow-up, two of the patients (7.41%) in the control group and all patients (100%) in the treatment group showed a reduction in PASI.

## 4. Discussion

Attention to the administration of probiotics for the treatment of psoriasis has recently increased. In the present study, improvement in DLQI, PASI, and VAS scores in the psoriasis patients receiving Lactocare® probiotics was compared to psoriasis patients receiving a placebo. In addition, the effect of age and sex on the efficacy of treatment on the improvement of these scores was evaluated. Multiple metrics such as PASI, DLQI, and VAS have been introduced to evaluate the efficacy of psoriasis treatment [21]. Currently, it has been demonstrated that the PASI, which combines the extent of the afflicted area and the assessment of the severity of lesions in a single index score, is the gold standard for determining therapy efficacy in patients with moderate-to-severe psoriasis [22]. The improvement of all three psoriasis indexes in the probiotic group at week 12 post-treatment compared to the placebo group was the main finding of the current study. On the other hand, at the 12-week follow-up, 100% of the patients in the treatment group and 7.41% of the patients in the control group both showed a reduction in PASI. These findings show that the effect of simultaneous administration of hydrocortisone with oral administration of probiotics on the improvement of psoriasis indexes is achieved 12 weeks after treatment. The improvement of the psoriasis index in the probiotic group may attribute to the anti-inflammatory effect and intestinal microbial composition of Lactocare®. There is no study conducted on these probiotics as a treatment in patients with psoriasis.

The inflammatory cytokines, such as TNF-*α*, IL-12, IL-23, and IL-17, are increased in the peripheral blood of psoriasis patients [5]. Phototherapy and topical or systemic immunosuppressants and anticytokine therapies are associated with a high economic cost and, rarely, they may activate latent infectious diseases [7, 8]. The elevated Th17 cells are notorious in the intestine and skin that are reported to relate to the pathogenesis of inflammatory diseases such as psoriasis. Th17 cells and their counterpart regulatory *T* cells are balanced by the intestinal microbiome [23]. Probiotics are nonpathogenic microorganisms with several potential mechanisms including adjustment of the intestinal microbiota composition, growth, function, and increasing local immune responses [24, 25]. The intestinal microbiota is the intestinal normal flora of the human body that plays an important role in human health, especially in the regulation of metabolic events and the development of the host immune system [24]. Lactocare®, a preprobiotic (synbiotic), has been used in this study that has been reported to treat mild-to-moderate ulcerative colitis due to its anti-inflammatory properties [17]. Despite the fact that exact pathogenesis of psoriasis is unknown, it is mostly believed that abnormal activation of immune cell functions plays an important role in the onset of the disease. Since psoriasis is an immune-mediated inflammatory disease, the control or stimulation of inflammatory response can improve or develop and progress disease [26].

Efficacy and safety of oral administration of probiotic strains and lower risk of relapse after the intake of the probiotic mixture in patients with psoriasis and modulation of the microbiota composition after 12 weeks have been recently reported by Navarro-López et al. [16]. IL-10, a type 2 cytokine, has been reported to have numerous immunosuppressive and anti-inflammatory properties. It has also inhibited antigen-presenting cells (APCs) including macrophages, dendritic cells, and monocytes [27–29]. Experimentally, administration of IL-10 resulted in shift-type 1 cytokines to type 2 cytokines. In addition, the treatment effect of ultraviolet light on the modulation of inflammation by up-regulation of the IL-10 in keratinocytes has been previously shown [30]. On the other hand, decreasing the level of IL-10 has been demonstrated to consider a critical way to induce disease flare-ups in the psoriatic skin [31].

In this study, the mean of PASI, DLQI, and VAS scores in the treatment group was also compared to the placebo group based on age and sex. According to these criteria, we have found no significant difference between male and female patients in the treatment group and the placebo group. Hägg et al. by measuring PASI in psoriasis patients reported that the severity of psoriasis differs between men and women [32]. This discrepancy can contribute to the number of patients and sample size or lifestyle such as smoking status and disease duration. However, the male-to-female ratio in our study was 1.73 indicating that men are more infected with psoriasis.

The obtained data from the intragroup analysis showed that the mean PASI, VAS, and DLQI scores were significantly decreased following spent time and treatment compared to the baseline in the treatment group, not the placebo. The highest decreasing PASI, VAS, and DLQI scores were detected 12 weeks after treatment followed by weeks 8 and 4. These findings show that the efficacy of the administration of probiotics in decreasing these scores can detect 4 weeks after treatment. In the present study, the age of 31 to 40 years old was reported as the highest frequency of age for having been infected with psoriasis. Middle-aged patients have been reported to show higher rates of severe nail psoriasis [33] which is in disagreement with our findings that may be due to the classification of age and sample size and the kind of disease in which they have only studied nail psoriasis.

## 5. Conclusion

In conclusion, PASI, DLQI, and VAS scores are improved in the psoriasis patients who were orally administered Lactocare® probiotic (two times daily) associated with topical administration of hydrocortisone 12 weeks after treatment. PASI reduction occurred in all patients who received probiotics. Oral administration of Lactocare® also could improve the psoriasis indexes 4 weeks after treatment. However, the disease was mostly detected in males, and the age between 31 and 40 years old was identified as the highest frequency age for infection.

### 5.1. Study Limitation

Since the study was conducted at the time of the COVID-19 pandemic, the lack of cooperation of all patients and the exclusion of some of them from the study was the main limitation of the present study.

## Figures and Tables

**Table 1:** Comparison of demographic data in both groups.

Variables	Treatment		Placebo		*p* value
	Frequency	Percent	Frequency	Percent	
Sex					
Male	16	64	17	62.96	0.93
Female	9	36	10	37.04	

*Continuous variables*					
	Mean	SEM	Mean	SEM	
Age	44.16	2.18	38.25	1.79	0.04
Duration	11.76	1.67	9.9	1.80	0.46

**Table 2:** Comparison of PASI, VAS, and DLQI scores in placebo and treatment groups at the baseline, weeks 4, 8, and 12.

Variables	Treatment	SEM	Placebo	SEM	*p* value
PASI baseline	7.22	0.61	6.47	0.42	0.31
VAS baseline	57.64	4.64	50.29	5.31	0.30
DLQ baseline	11.88	0.90	9.81	1.09	0.15
PASI week 4	6.7	.60	6.44	0.41	0.72
VAS week 4	50.44	4.51	49.51	5.18	0.89
DLQI week 4	10.76	0.83	9.70	1.01	0.42
PASI week 8	5.67	0.49	6.33	0.39	0.29
VAS week 8	41.80	4.43	48.51	4.94	0.32
DLQI week 8	8.96	0.73	9.40	0.86	0.69
PASI week 12	4.08	0.28	6.19	0.39	<0.001
VAS week 12	32.24	4.29	48.11	4.88	0.019
DLQI week 12	6.56	0.63	8.92	0.71	0.017

**Table 3:** Comparison of PASI, VAS, and DLQI scores in the treatment group at the baseline, weeks 4, 8, and 12.

Variable/time	Baseline	week 4	week 8	week 12
PASI	7.22 ± 0.61a	6.7 ± 0.60b	5.67 ± 0.49c	4.08 ± 0.39d
VAS	57.64 ± 4.64a	50.44 ± 4.51b	41.8 ± 4.43c	32.24 ± 4.29d
DLQI	11.88 ± 0.90a	10.76 ± 0.83b	8.96 ± 0.73c	6.56 ± 0.63d

The difference letters in each row show significant difference at *p* < 0.05.

**Table 4:** Comparison of PASI, VAS, and DLQI scores in each group at the baseline, weeks 4, 8, and 12.

Variable/time	Baseline	week 4	week 8	week 12
PASI	6.47 ± 0.42a	6.44 ± 0.41a	6.33 ± 0.39a	6.19 ± 0.39a
VAS	50.29 ± 5.31a	49.51 ± 5.18a	48.51 ± 4.94a	48.11 ± 4.88a
DLQI	9.81 ± 1.09a	9.70 ± 1.01a	9.4 ± 0.86a	8.92 ± 0.71a

The difference letters in each row show significant difference at *p* < 0.05.

**Table 5:** The PASI, VAS, and DLQI scores of various ages.

	Treatment group			
Variable/age	20–30	31–40	41–50	Up to 51
PASI baseline	9.94 ± 1.73	8.17 ± 2.21	6.22 ± 0.57	6.04 ± 0.77
PASI week 4	9.46 ± 1.75	7.77 ± 2.21	5.67 ± 0.56	5.42 ± 0.61
PASI week 8	7.80 ± 1.20	6.70 ± 1.95	4.98 ± 0.50	4.44 ± 0.47
PASI week 12	5.14 ± 0.47	4.50 ± 1.11	3.82 ± 0.42	3.42 ± 0.35
VAS baseline	64.00 ± 5.09	57.50 ± 12.50	59.11 ± 8.80	51.28 ± 10.30
VAS week 4	57.60 ± 5.59	52.00 ± 11.53	52.11 ± 8.84	42.28 ± 9.29
VAS week 8	52.00 ± 6.34	41.75 ± 12.37	44.55 ± 8.43	31.00 ± 7.89
VAS week 12	44.40 ± 7.33	31.75 ± 11.52	34.33 ± 8.26	21.14 ± 6.62
DLQI baseline	12.60 ± 0.74	14.75 ± 3.01	12.00 ± 1.71	9.57 ± 1.42
DLQI week 4	11.40 ± 0.5	13.75 ± 2.65	11.00 ± 1.54	8.28 ± 1.32
DLQI week 8	10.20 ± 0.48	10.50 ± 2.10	9.00 ± 1.59	7.14 ± 10.1
DLQI week 12	8.20 ± 0.78	7.75 ± 1.22	6.66 ± 1.79	4.57 ± 0.96

Control group				

PASI baseline	7.94 ± 0.91	5.87 ± 0.46	6.14 ± 1.34	7.02 ± 1.37
PASI week 4	7.94 ± 0.91	5.800 ± 0.49	5.98 ± 1.18	7.02 ± 1.37
PASI week 8	7.94 ± 0.91	5.80 ± 0.49	5.58 ± 0.81	7.02 ± 1.37
PASI week 12	7.94 ± 0.91	5.73 ± 0.51	4.96 ± 0.38	7.02 ± 1.37
VAS baseline	51.00 ± 10.29	47.50 ± 8.66	50.00 ± 14.23	60.50 ± 10.69
VAS week 4	50.40 ± 10.12	46.46 ± 8.56	48.20 ± 13.10	60.00 ± 10.63
VAS week 8	50.40 ± 10.12	45.84 ± 8.34	44.40 ± 10.51	60.00 ± 10.63
VAS week 12	50.40 ± 10.12	45.61 ± 8.26	42.80 ± 9.63	60.00 ± 10.63
DLQI baseline	8.60 ± 1.50	10.15 ± 1.96	8.80 ± 2.17	11.50 ± 2.32
DLQI week 4	8.60 ± 1.50	9.92 ± 1.77	8.80 ± 2.17	11.50 ± 2.32
DLQI week 8	8.60 ± 1.50	9.92 ± 1.77	8.80 ± 2.17	11.50 ± 2.32
DLQI week 12	8.60 ± 1.50	8.79 ± 1.09	7.60 ± 1.12	11.50 ± 2.32

## Data Availability

All data are available via the corresponding author.

## Additional Points

*Impact of Findings on Practice Statements*. (i) Probiotics are important in keeping the body healthy. (ii) Oral administration of probiotics improves symptoms in patients with psoriasis.

## Supplementary Materials

Supplementary Materials Supplementary. 1: the mean PASI score in the treatment (*A*) and placebo (B) groups at the baseline (0), weeks 4 (1), 8 (2), and 12 (3) post-treatment. Supplementary. 2: the mean VAS score in the treatment (*A*) and placebo (B) groups at the baseline (0), weeks 4 (1), 8 (2), and 12 (3) post-treatment. Supplementary. 3: the mean DLQI score in the treatment (*A*) and placebo (B) groups at the baseline (0), weeks 4 (1), 8 (2), and 12 (3) post-treatment. Supplementary 4: comparison of PASI, VAS, and DLQI scores at the baseline and week 12 post-treatment in female and male patients in both groups.
