# Supplementary material for: The Efficacy of Lactocare® Synbiotic on the Clinical Symptoms in Patients with Psoriasis: A Randomized, Double-Blind, Placebo-Controlled Clinical Trial
Source: Dermatol Res Pract. 2022 Oct 7;2022:4549134. doi: 10.1155/2022/4549134 (PMC9568340; doi:10.1155/2022/4549134)
Supplement: Supplementary Materials — Supplementary. 1: the mean PASI score in the treatment (A) and placebo (B) groups at the baseline (0), weeks 4 (1), 8 (2), and 12 (3) post-treatment. Supplementary. 2: the mean VAS score in the treatment (A) and placebo (B) groups at the baseline (0), weeks 4 (1), 8 (2), and 12 (3) post-treatment. Supplementary. 3: the mean DLQI score in the treatment (A) and placebo (B) groups at the baseline (0), weeks 4 (1), 8 (2), and 12 (3) post-treatment. Supplementary 4: comparison of PASI, VAS, and DLQI scores at the baseline and week 12 post-treatment in female and male patients in both groups. [file 4549134.f1.docx]

**Supplementary files**

**Supplementary. 1**: The mean PASI score in the treatment (A) and placebo (B) groups at baseline (0), weeks 4 (1), 8 (2), and 12 (3) post-treatment


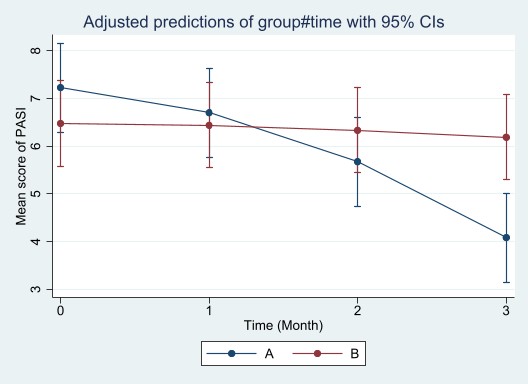


**Supplementary. 2**: The mean VAS score in the treatment (A) and placebo (B) groups at baseline (0), weeks 4 (1), 8 (2), and 12 (3) post-treatment


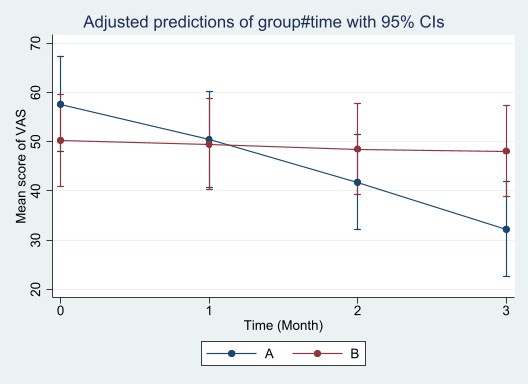


**Supplementary. 3**: The mean DLQI score in the treatment (A) and placebo (B) groups at baseline (0), weeks 4 (1), 8 (2), and 12 (3) post-treatment


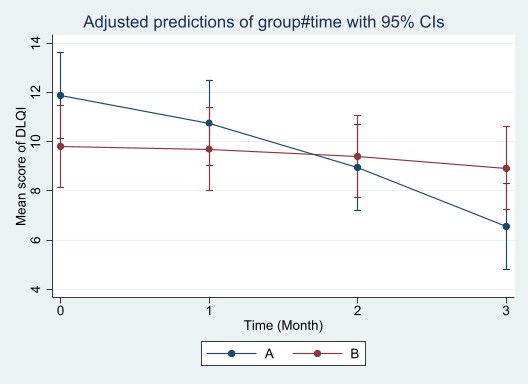


**Supplementary** **4**. Comparison of PASI, VAS, and DLQI scores at baseline and week 12 post-treatment in female and male patients in both groups

| **Variable/ sex** | | **Treatment** | **P value** | **Placebo** | **P value** |
| --- | --- | --- | --- | --- | --- |
| PASI baseline | Male | 6.88±0.87 | 0.30 | 6.52±0.55 | 0.71 |
|  | Female | 7.84±0.71 |  | 6.40±0.69 |  |
| PASI week 4 | Male | 6.36±0.86 | 0.32 | 6.51±0.55 | 0.73 |
|  | Female | 7.28±0.75 |  | 6.32±0.63 |  |
| PASI week 8 | Male | 5.31±0.64 | 0.27 | 6.46±0.56 | 0.75 |
|  | Female | 6.31±0.73 |  | 6.12±0.51 |  |
| PASI week 12 | Male | 3.86±0.38 | 0.18 | 6.41±0.58 | 0.22 |
|  | Female | 4.47±0.34 |  | 5.81±0.44 |  |
| VAS baseline | Male | 53.62±6.36 | 0.28 | 47.76±7.00 | 0.24 |
|  | Female | 64.77±5.88 |  | 54.60±8.28 |  |
| VAS week 4 | Male | 46.37±5.98 | 0.26 | 47.23±6.86 | 0.31 |
|  | Female | 57.66±6.35 |  | 53.40±8.04 |  |
| VAS week 8 | Male | 38.06±5.79 | 0.27 | 46.76±6.70 | 0.54 |
|  | Female | 48.44±6.57 |  | 51.50±7.32 |  |
| VAS week 12 | Male | 28.81±5.44 | 0.31 | 46.58±6.64 | 0.53 |
|  | Female | 38.33±6.91 |  | 50.70±7.14 |  |
| DLQI baseline | Male | 10.81±1.11 | 0.17 | 9.11±1.59 | 0.22 |
|  | Female | 13.77±1.40 |  | 11.00±1.17 |  |
| DLQI week 4 | Male | 9.81±1.03 | 0.18 | 8.94±1.44 | 0.20 |
|  | Female | 12.44±1.28 |  | 11.00±1.17 |  |
| DLQI week 8 | Male | 8.31±0.94 | 0.21 | 8.58±1.19 | 0.19 |
|  | Female | 10.11±1.13 |  | 10.80±1.07 |  |
| DLQI week 12 | Male | 6.00±0.83 | 0.26 | 8.05±0.94 | 0.19 |
|  | Female | 7.55±0.89 |  | 10.40±0.96 |  |
